# Validation of monoclonal anti-PKC isozyme antibodies for flow cytometry analyses in human T cell subsets and expression in cord blood T cells

**DOI:** 10.1038/s41598-019-45507-2

**Published:** 2019-06-25

**Authors:** Khalida Perveen, Alex Quach, Andrew McPhee, Susan L. Prescott, Simon C. Barry, Charles S. Hii, Antonio Ferrante

**Affiliations:** 1 Department of Immunology, SA Pathology at Women’s and Children’s Hospital, North Adelaide, South Australia Australia; 2 The Robinson Research Institute and School of Medicine, University of Adelaide, South Australia, Australia; 3 Department of Neonatal Medicine, Women’s and Children’s Hospital, North Adelaide, South Australia Australia; 4 Department of Paediatrics, University of Western Australia, Western Australia, Australia

**Keywords:** T-helper 1 cells, Allergy

## Abstract

T cells from neonates (cord blood) with a tendency to develop allergic diseases express low PKCζ levels. More extensive investigations into PKC isozyme levels in T cell subsets and changes during neonatal T cell maturation are hampered by limitations of Western blot analyses. We have undertaken to validating the specificity of commercially available antibodies marketed for flow cytometry to measure PKCα, βI, βII, δ, ε, η, θ, ζ, ι/λ and μ. Western blot analyses of human peripheral blood mononuclear cell (PBMC) lysates demonstrated that some antibodies were unsuitable for flow cytometry assays. A panel of antibodies with the desirable specificity and reliability in the flow cytometry assay were identified using both PBMC and whole blood assays. The results showed that all PKC isozymes were expressed in CD4^+^ and CD8^+^ T cells, monocytes and neutrophils. Murine lymphocytes showed similar patterns of expression. A major finding was that 35.2% and 38.5% of cord blood samples have low PKCζ (≤the 5^th^ percentile of adult levels) in the CD4^+^ and CD8^+^ subsets, respectively, consistent with the incidence of allergy development in the population. Furthermore, these low PKCζ levels ‘normalised’ within 24 h after initiation of maturation of these cells in culture, providing a ‘window of opportunity’ for altering PKCζ levels.

## Introduction

Protein kinase Cs (PKCs) are a family of phospholipid-dependent serine/threonine protein kinases which consists of at least 11 isoforms, each with tissue-specific distribution and individual functional properties. On the basis of structure, requirement for Ca^2+^ for activation and unique binding ability with phorbol myristate acetate (PMA) or diacylglycerol (DAG), they have been divided into three groups: classical or conventional PKC (consisting of PKCα, βI, βII and γ), novel (PKCδ, ε, η and θ) and atypical (PKCζ, ι/λ and μ) isozymes^1^. The role of PKC isozymes in cell proliferation, differentiation^1^, motility and survival^2^ has been well established. Metabolic disorders^3^, immune immaturity^4^, cancer^5,6^ and cardiovascular malfunction^7^ are some of the diseases that have been associated with mutation/altered expression of specific PKC genes. Differences in levels of PKCs or the abnormal activation have been reported in autoimmune diseases, heart failure, acute and chronic heart disease, kidney and lung diseases, diabetes, various dermatological diseases, psychiatric diseases, cancer as well as neurological conditions^8^. Involvement of PKC in these abnormalities highlights the critical role that these kinases perform in cell signalling and regulation of health and disease development.

Reduced T cell PKCζ levels are associated with Th2 dominance at birth and the subsequent failure to develop towards T helper (Th)1 phenotype whereas high T cell PKCζ expression at birth was associated with non-allergy development in infants^9–12^. The studies also showed that the levels of PKCζ in cord blood T cells (CBTC) could be used as a biomarker for assessing whether children were likely to develop allergic diseases/sensitisation^10,11^. This notion was further supported by the findings that supplementation with omega3 fats during pregnancy increased the levels of PKCζ in CBTC and this was associated with reduced allergic disease development and a decrease in Th2 persistence in maturing cells^10–12^. Furthermore the changes in PKCζ expression induced by omega3 fats were under an epigenetic control^12^. These findings provide a new concept in the regulation of disease development from birth. To gain more insights into this concept we have now attempted to validate flow cytometry assays for the range of PKC isozymes and used this to evaluate the levels in the different leukocyte types and lymphocyte subsets and explored their levels in neonatal leukocytes, at birth and during T cell maturation.

Here we demonstrate the need to ensure specificity of antibodies designed to be used for flow cytometry assays to measure intracellular levels of PKC isozymes. Following validation of their specificity and adaptation to flow cytometry assays, we demonstrated the presence of PKCα, βI, βII, δ, ε, η, θ, ζ, ι/λ and μ in T cell subsets, monocytes and neutrophils. These measurements could be made in whole blood assays. The antibodies were also able to detect all of these PKC isotypes in mouse splenic and peripheral blood lymphocytes. Examination of PKC isozyme levels in CBTC demonstrated that 30–40% (of the cord bloods) were deficient in PKCζ, a deficiency rate expected for high risk of allergy development in the population. Furthermore, we were able to show that these low levels rapidly normalise following the initiation of CBTC maturation in a culture model system.

## Results

### Specificity of anti-PKC isozyme antibodies

**Figure 1:**
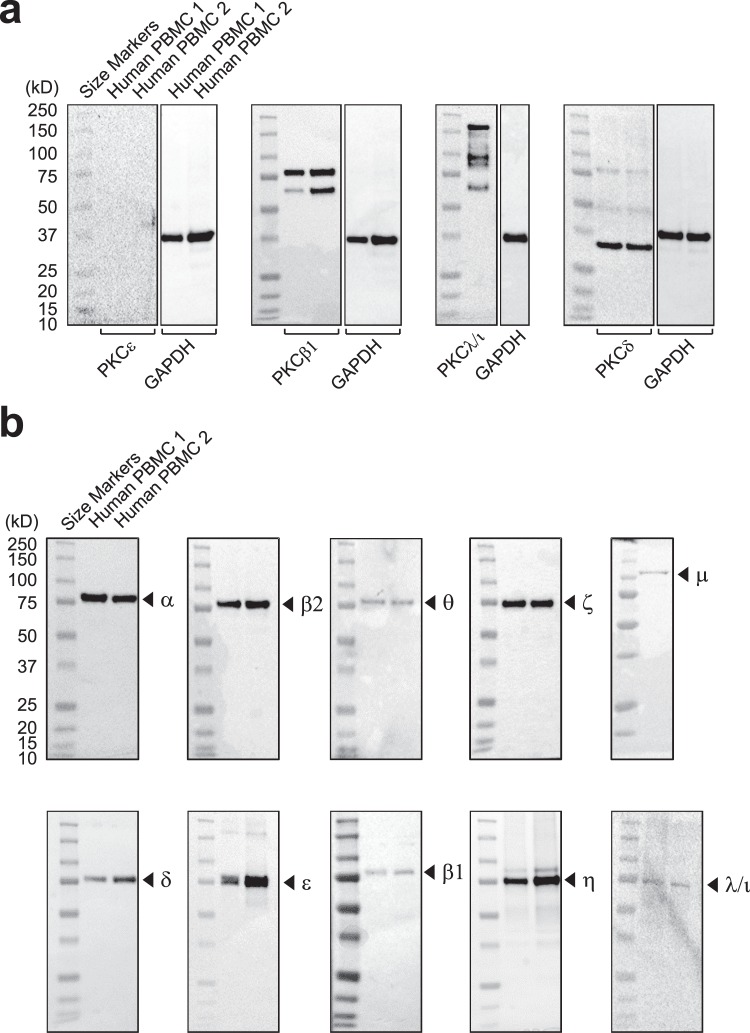
Specificity of the anti-PKC isozyme monoclonal antibodies. Lysates from PBMC from human blood of adult volunteers were subjected to Western blot analysis using isoenzyme-specific monoclonal anti-PKC antibodies as indicated in figures. These were developed with an HRP-conjugated anti-mouse/anti-rabbit Ig antibody. (**a**) Shows the reactivity of antibodies recommended for flow cytometry. The blots show either one or two samples (lanes 1 and 2) with blots stripped and reprobed to detect GAPDH. These antibodies either did not show any immunoreactivity towards the intended PKC isozyme or they showed non-specific binding to multiple proteins. Antibodies: anti-PKCε, -PKCβI, -PKCλ/ι and -PKCδ antibodies were from clones E-5, E-3, E-7 and G-9; (Supplementary Table S1). (**b**) Specificity of antibodies selected for flow cytometry assay. These anti-PKC antibodies were selected based on their specificities, as they each showed specific binding to a single protein band. The clones for the anti-PKCα (H-7), βI (EPR18512), βII (F-7), δ (EPR17075), ε (EPR1482(2)), η (EPR18513), θ (E-7), ζ (H-1), ι/λ (H-12) and μ (EP1493Y) are shown in parentheses (see also Supplementary Table S1). The blots are presented individually for each staining, intact without splicing, with lanes in their entirety.

To validate the specificity of commercially available anti-PKC isozyme antibodies for flow cytometry, we examined their specificities against their respective targets by Western blot using lysates from human PBMC. Four of the antibodies (anti-PKCε, -PKCβI, -PKCλ/ι and -PKCδ antibodies; clones E-5, E-3, E-7 and G-9; see Supplementary Table S1) that were designated for use in Western blot and flow cytometry assays were found not to be suitable for the flow cytometry assay (Fig. 1a). The anti-PKCε antibody was not able to detect the PKC isozyme in these lysates; that against PKCIβ detected an additional protein and the anti- PKCλ/ι showed several protein bands. The antibody to PKCδ also detected other proteins apart from this PKC isozyme. While these antibodies have been recommended for use in both Western blot and flow cytometry, it is evident that for human leukocytes they are not appropriate for use in the latter.

After further examination, we identified ten antibodies that had the specificities needed for the detection and estimation of PKCα, βI, βII, δ, ε, η, θ, ζ, ι/λ or μ by flow cytometry (clones outlined in Fig. 1 legend). The data in Fig. 1b show the Western blot analyses of PBMC lysates probed with these antibodies. All isozymes were detected at their expected molecular weights, around 80 kDa, except for PKCμ that migrated in SDS-PAGE gels at the expected 115 kDa. In all cases there was a high level of specificity for each of these isozymes.

### Flow cytometry validation of the PKC isozyme measurements in whole blood assays

**Figure 2:**
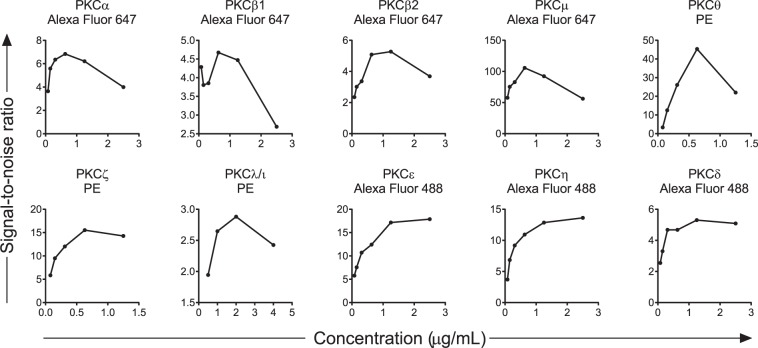
Signal-to-noise ratios of titrated anti-PKC isozyme antibodies in whole blood assay of lymphocytes. The lymphocyte fraction of whole blood was gated and assessed for staining by the anti-PKCα, βI, βII, θ, δ, ε, ζ, λ/ι μ and η antibodies along with the appropriate fluorochrome-congujated isotype controls. Signal-to-noise ratios were calculated as described in the methods. The final concentration of each antibody with the respective isotype control is indicated in the graphs. The MFI graphs are shown in Supplementary Fig. S2.

The above Western blot-validated antibodies were then examined for suitability in flow cytometry assays for detecting intracellular PKC isozymes. Anti-PKCα, βI, βII and μ (AF647 conjugated) and PKCη, ε and δ (AF488) and PKCθ, ζ and λ/ι (PE) conjugated antibodies were titrated in parallel with the isotype control. Supplementary Fig. S1 shows the gating strategies. Data for the titration of the anti-PKC antibodies is presented as signal-to-noise ratios in Fig. 2, with the MFI presented in Supplementary Fig. S2. The results showed that lymphocytes stained for all the PKC isozymes in a concentration related manner.

**Figure 3:**
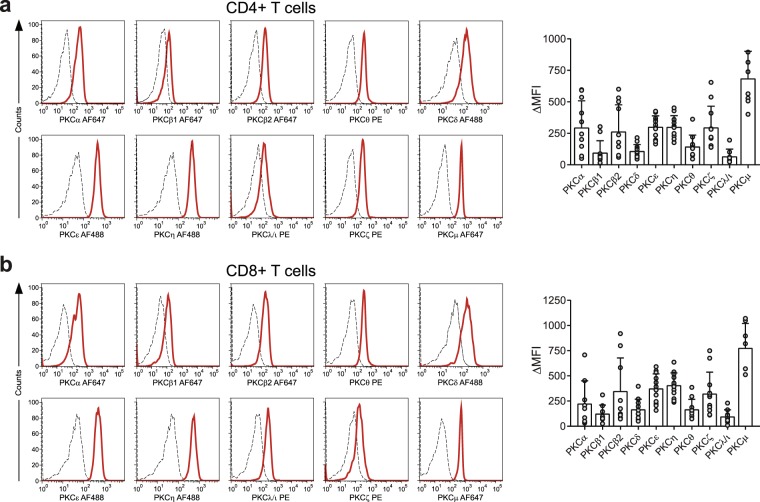
Expression of PKC isozymes in CD4^+^ and CD8^+^ T cells by flow cytometry analysis. Flow cytometry analysis of PKC isozyme expression in CD4^+^ and CD8^+^ T cells. (**a**) Representative histogram for PKC isozymes in whole blood assay gated on CD3^+^CD8^−^ T-cells (left) and individual and mean values showing the variation in the population (n = 11) (right). (**b**) Representative histogram for PKC isozymes in whole blood assay gated on CD3^+^CD8^+^ T cells and values representing the variation in the population (n = 11). Dashed lines represent the isotype controls and solid lines represent anti-PKC isozyme antibody staining. Bar graphs show values for each individual and the mean ± SD expressed as change in median fluorescence intensity (ΔMFI) obtained after subtracting the isotype control MFI values from respective PKC isozyme MFI values.

**Figure 4:**
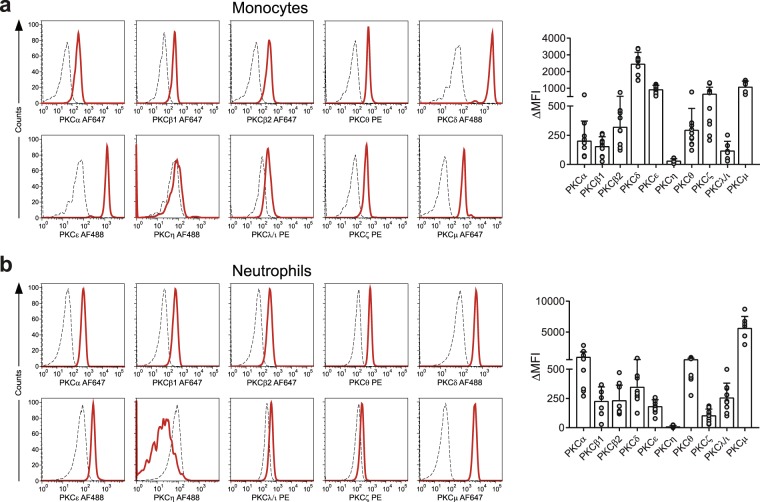
Expression of PKC isozymes in monocytes and neutrophils using whole blood flow cytometry assays. Intracellular staining was performed on fresh whole blood as per method section. Representative histogram for PKC isozymes in whole blood assay gated on monocytes (**a**) or neutrophils (**b**), showing also values for the population. Dashed lines represent the isotype control and solid lines represent the respective PKC isozyme in each cell population. Analysis of each PKC isozyme was performed by gating the subpopulation based on their light scatter patterns for monocytes and neutrophils. Bar graphs represent values for each individual and as mean ± SD, expressed as change in median fluorescent intensity (ΔMFI) which were obtained after subtracting the isotype control MFI values from respective PKC isozymes MFI values. Sample size = 7–11 samples for neutrophils and monocytes.

**Figure 5:**
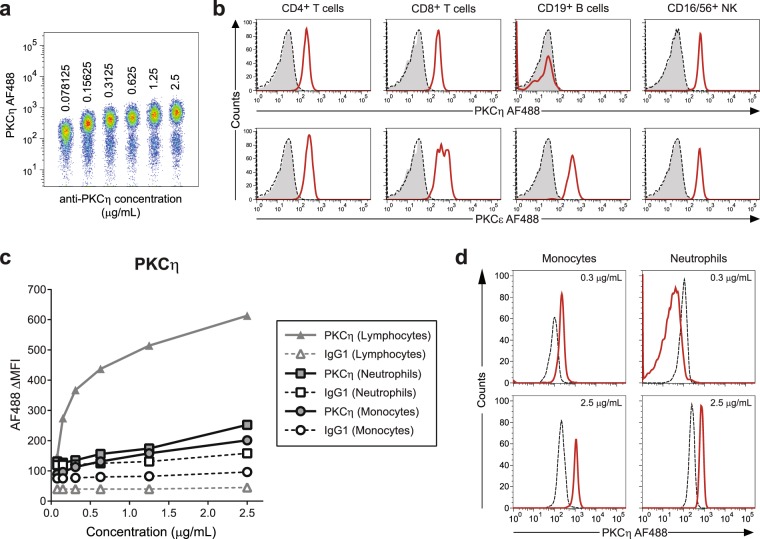
Expression of PKCη in lymphocyte subsets, neutrophils and monocytes, assessed by flow cytometry in whole blood. (**a**) Titration of anti-PKCη antibody. Intracellular staining of cells was performed and the fluorescence signal in the lymphocyte gate was analysed. (**b**) PKCη in lymphocyte subsets. Whole blood was surface stained for CD4^+^ T cells, CD8^+^ T cells, natural killer cells (CD16/56) and B cells and then stained for intracellular PKCη expression. PKCε was used as a positive control. Shaded histograms represent the unstained population, dashed and solid lines represent the isotype and PKC expression, respectively (n = 3). (**c**) Titration results in neutrophils and in monocytes using lymphocytes as the positive control. (**d**) Histograms for PKCη expression in monocytes and neutrophils at two antibody concentrations. Dashed lines represent the isotype control, solid lines represent the PKCη expression (n = 2).

The data in Fig. 3 show the expression of the various PKC isozymes in both CD4^+^ and CD8^+^ T cells from healthy donors. There was a wide variation in levels of specific isozymes expressed between these individuals. However, the levels expressed in the different isozymes were similar between the two T cell subsets (Fig. 3). Further analyses on the monocyte and neutrophil populations showed also the expression of all the isotypes in these cells (Figs 4 and 5).

A small proportion of lymphocytes lacked PKCη (Fig. 5a). Further, analysis for PKCη in CD4^+^ and CD8^+^ T cells, B cells and NK cells revealed that these all expressed the PKC isozymes except for B cells which lacked PKCη (Fig. 5b). Examination of neutrophils and monocytes for all PKC isozymes revealed that neutrophils and monocytes express all PKC isozymes (Fig. 4; Fig. S3). The data displayed in Supplementary Fig. S3 show the differential expression of these isozyme in different leukocyte subpopulations. Thus PKCα, ι/λ, θ and μ are the most highly expressed in neutrophils, PKCδ and ε in monocytes and PKCη in T cells.

### Analysis of PKC isozymes in murine lymphocytes

**Figure 6:**
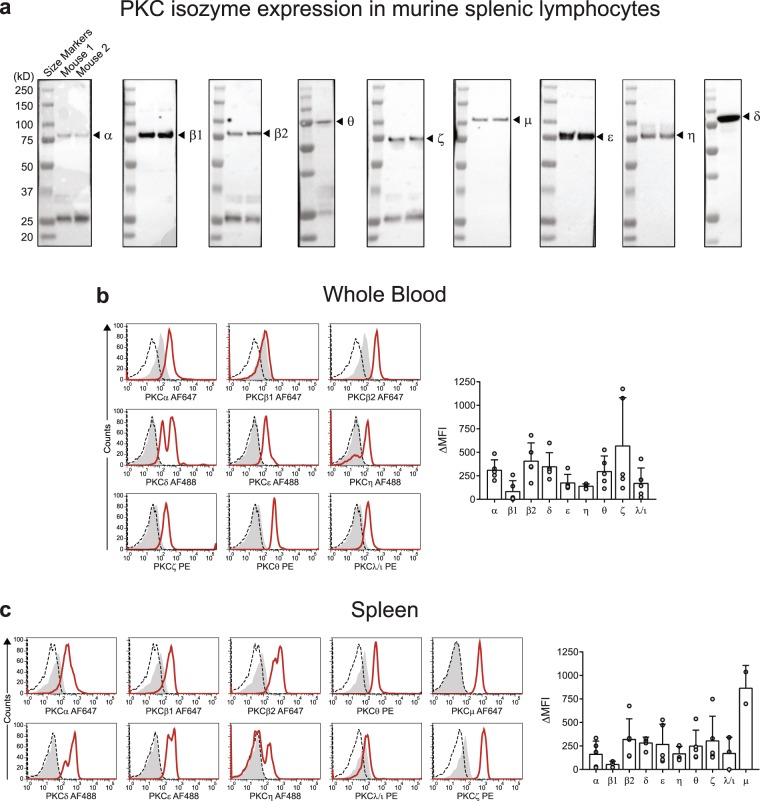
Detection of PKC isozyme expression in mouse lymphocytes by Western blot and flow cytometry. Mouse spleens were removed and cells were separated. (**a**) Cell lysates were prepared for Western blot analysis for detection of PKC isozymes by using PKC isozymes specific antibodies as shown in figures, *n* = 1–2. The blots are presented individually for each staining, intact without splicing, with lanes in their entirety. (**b**) Murine whole blood or (**c**) splenic lymphocytes were stained for intracellular PKC isozymes expression as outlined in methods. Histogram data from murine lymphocyte population are shown. Shaded histogram represents isotype control, dashed line represents unstained population and solid line represents PKC isozymes. Quantitated data (mean ± SD) from mouse blood lymphocytes (*n* = 5) and mouse splenic cells (*n* = 2–5) are presented in the right hand panels.

The analyses were extended to expression of the PKC isozymes in murine lymphocytes. The above antibodies specific for the PKC isozymes were used to examine their expression in murine splenic lymphocytes, using outbred Swiss white mice. Examination of lysates by Western blotting revealed a similar level of specificity (Fig. 6a) as for the human PBMC (Fig. 1). The unidentified lower molecular weight bands of approximately 25–35 kDa in the PKCα, βII, θ and ζ blots were unlikely to be due to degraded PKC or to non-specific staining by the primary antibodies since it was also detected in the absence of a primary antibody (Supplementary Fig. S4), suggesting that it is due to the secondary antibody. However, this would not have any implications in the flow cytometry assay which is based on direct staining of the PKC isozymes.

Flow cytometry analysis was conducted as per human PBMC for detection of the different isozymes in splenic lymphocytes as well as in whole blood assays. As we were only interested in whether these antibodies were able to detect the isozymes in murine lymphocytes by flow cytometry assay, no subset characterisation was undertaken. The analysis was performed by SSC and FSC properties of murine cells, after exclusion of doublets by FSC-H and FSC-A. All PKC isozymes were detected in murine lymphocytes from blood and splenic cells (Fig. 6b,c). Furthermore, like human leukocytes, mouse blood lymphocytes and splenic cells showed two distinct populations based on presence or absence of PKCη (Fig. 6b,c). A more prominent population of PKCη negative was seen in splenic cells than blood since the former contain ~50% B cells.

### Levels of PKC isozymes in cord blood CD4^+^ and CD8^+^ T cells, monocytes and neutrophils

**Figure 7:**
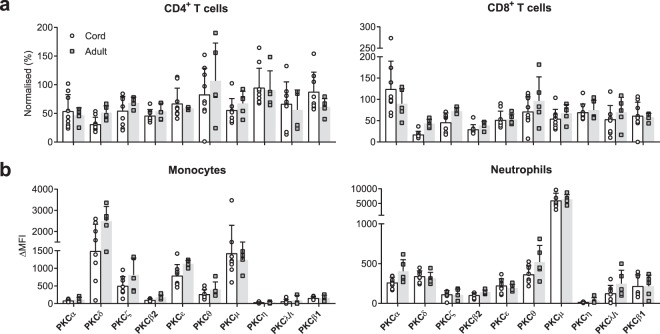
Cord blood PKC isozyme expression in CD4^+^ and CD8^+^ T cells, monocytes and neutrophils. Whole blood from cord (n = 9) and adult donor blood (n = 4) were stained with anti-CD3 and anti-CD8 antibodies and co-stained for anti-PKC isozymes. The data are expressed as a percentage of cryopreserved adult donor PBMC that were analysed in parallel. PKC levels were quantified as change in median fluorescent intensity (ΔMFI) by flow cytometry and then expressed as percentage of cryopreserved adult control cells. (**a**) Expression of PKC isozymes in cord (open bars) and adult (shaded bars) blood CD4^+^ and CD8^+^ T cells. (**b**) Expression of PKC isozymes in cord blood monocytes and neutrophils as compared to adult control blood. These levels were quantified as change in ΔMFI after subtracting the MFI of respective isotype control by flow cytometry and shown as median. Data are presented as mean ± SD.

Previously, we have examined, by Western blot analyses, the levels of a limited number of PKC isozymes in T cells from cord bloods of subjects with a family history of allergic diseases^11^. Here we have used a non-selected population of cord bloods to examine the levels by flow cytometry. Adult donor blood and cryopreserved PBMC from healthy adult donors were run in parallel as quality assurance for the assay. CB samples were analysed for PKC isozymes expression in CD4^+^ and CD8^+^ cell populations. The results showed that for both of these a proportion of the cord bloods displayed low PKC**ζ** and δ (Fig. 7a). We also show that these were deficient in monocytes but this was less evident in neutrophils (Fig. 7b).

### Changes in PKC isozyme levels in cord blood T cells during maturation in culture

**Figure 8:**
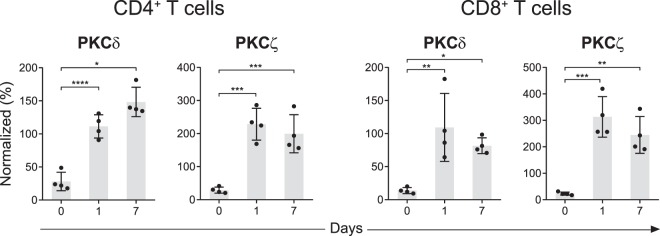
Kinetics of PKCζ and δ expression in cord blood CD3^+^CD8^−^ and CD3^+^CD8^+^ T cells during maturation in culture. Cord blood T cell subsets expressing low levels of PKCζ at day-0 were matured in the presence of PHA (2 µg/ml), and IL-2 (10 ng/ml). Levels of the PKC isozymes were analysed by intracellular staining method as per method section at the indicated time point in (**a**) CD4^+^ and (**b**) CD8^+^ T cells during culture. ΔMFI of PKCs expressed as percentage of ΔMFI in cryopreserved PBMCs (n = 4). *P < 0.05, **P < 0.01, ***P < 0.001, ****P < 0.0001.

In this aspect of the study we selected those CBTC which showed low or deficient levels of PKCζ. These low levels were based on our normal range (5^th^-95 percentiles) of 35.2–90.5% for CD4^+^ T cells and 38.5–92.3% for CD8^+^ T cells. Since PKC is important in T cell responses once the cells have matured, it was expected that those with low levels would normalise during the maturation process. Using a previously described *in vitro* T cell maturation system^9–13^, we examined whether the PKC isozyme levels normalized in those cord cells which expressed low levels. The data presented in Fig. 8a for CD4^+^ T cells and Fig. 8b for CD8^+^ T cells, show that within 24 h of the commencement of the maturation process, the PKC isozymes had already increased to mature T cell levels, with no further increases after this time (Fig. 8).

## Discussion

While it has been reported that low levels of PKCζ in CBTC may be a risk factor for development of allergy, the acceptance of this phenomenon awaits more profound studies. This includes the relationship in expression of this isozyme to other PKC isozymes, expression in CD4^+^ and CD8^+^ T cells and kinetics of normalisation of the levels during CBTC maturation. Advancements can be made if levels of the PKC isozymes can be measured by flow cytometry, together with the characterization in T cell subsets and with assessing of cytokine production. To facilitate such studies, we have shown that translating assays from Western blot to flow cytometry requires validation of these antibodies. Our data show that despite claims that monoclonal antibodies are suitable for flow cytometry, we have shown that when tested on Western blot some of them may not be specific for the indicated PKC isozymes. By subjecting the antibodies to Western blot analysis we were able to identify a panel which could specifically detect the PKC isozymes. This enabled the identification of levels of these isozymes in human lymphocytes, T cell subsets, B cells, NK cells, monocytes and neutrophils in whole blood assays.

The advantages of using flow cytometry are evident from the finding that we detected a PKCη positive and negative populations in peripheral blood lymphocytes population. It was evident that CD4^+^, CD8^+^ and NK cells are positive for PKCη, while B cells lack this PKC isozyme in both human and mice. This has been reported in developing B cells, which expressed high PKCη at mRNA level, while mature or resting B cells have low levels of PKCη^14^. This finding is consistent with our data from flow cytometry studies.

Overall, all PKC isozymes were detectable in CD3^+^ T cells in whole blood assays. The data show that levels of each PKC isozyme in CD4^+^ and CD8^+^ T cell populations were comparable. The low expression of PKCβI in human monocytes is consistent with the literature^15^.

PKC has been reported to play various roles in T cell function. The atypical PKCζ and PKCλ/ι play a role in regulation of asymmetric CD8^+^ T cell division involving the differentiation of CD8^+^ T cells to a long-lived effector phenotype with reduced memory T cell development^16^. PKCθ plays an important role in Th2 cell type development but has no effect on Th1 cell responses^17^. PKCε is important for CD4 T cell proliferation^18^.

While PKC isozyme expression in monocytes is well documented, by Western blot analysis, their expression in neutrophils remains either incompletely characterized or discordant. In rat neutrophils, Tsao and Wang^19^ reported the expression of PKCα, β, δ, ε, θ, μ, ι, λ, ζ and γ (with γ being normally detected in neuronal cells) albeit at various levels. In contrast, Dang, *et al*.^20^ examined the expression of six PKC isozymes in human neutrophils and was unable to detect PKCδ, ε and γ. Balasubramanian, *et al*.^21^ examined the expression of all PKC isozymes in human neutrophils and were able to detect all except PKCε, ζ, γ and η. The basis for these differences is unclear even though an argument based on species differences could be made for the findings of Tsao and Wang^19^ and those of Balasubramanian, *et al*.^21^ as both groups used antibodies from the same commercial source. We optimised our flow cytometry-based assay on whole blood to examine their expression in these cells. All PKC isozymes are expressed in monocytes and neutrophils. All PKC isozymes were detectable in mouse lymphocytes from blood and splenic cells.

Using the flow cytometry assay, a number of cord blood samples were examined for PKC expression in a random population. It was evident from these results that only about 40% of these were considered to be low or deficient in PKCζ compared to adult control values. This was evident in both CD4^+^ and CD8^+^ T cells. Although we previously only examined the CD3^+^ T cell population, it was evident that a high proportion of that cohort, which comprise babies born to women who had a family history of allergic diseases, displayed low expression of PKCζ^10^. Our present findings provide further support to the proposal that PKCζ expression levels in T cells are prognostic for risk of allergy development since it has been claimed that 40% of the population are at risk of developing allergic diseases^22^. Interestingly, the only other isozyme found to be deficient in cord blood CD4^+^ and CD8^+^ T cells was PKCδ. Previously we have not found the levels of this isozyme to be correlated with development of allergic diseases^11^. Amongst the other leukocyte subpopulations neutrophils did not show the PKC isozyme deficiency but monocytes from some cord bloods presented with deficient isozymes, PKCδ and PKCζ. It will be interesting to follow up the consequence of these low levels in some cord blood monocytes. Thus the isozyme levels can be determined in whole blood without the need to purify the subpopulations of leukocytes.

Examination of PKCζ and δ during CD4^+^ and CD8^+^ T cell maturation in the culture model showed that when fully matured, both isotypes were expressed at mature T cell levels. In fact, their expression normalised within 24 h of initiating maturation, and before the expression of maturation markers CD45RA^−^/RO^+^. Our previously published data demonstrated that PKCζ is required to prevent cells from maintaining an immature Th2 allergic cytokine profile, a characteristic of neonatal T cells^12^. Taken together these results lead to an understanding that not only PKCζ may be a biomarker for propensity to develop allergic diseases but may also have a functional role in development of T cells towards a non-allergic cytokine profile. The significance of our finding also extends to identification of an early period during which this development can be ‘re-programmed’^11,12^.

## Materials and Methods

### Reagents, chemicals and antibodies

Information about the antibodies for intracellular staining for PKC isozymes, leukocyte surface markers and the labelling kit used for antibody-fluorescent dye conjugation isotype controls and mouse and rabbit IgG blocking antibodies, are summarized in Supplementary Table S1. RPMI 1640 tissue culture medium, foetal calf serum (FCS) and L-glutamine were purchased from SAFC Biosciences (Lenexa, Kansas, USA).

### Ethics statement

The procurement of human blood and all experimental procedures were approved by the Human Research Ethics Committee of the Women’s and Children’s Health Network (WCHN), Adelaide, South Australia, in accordance to The *National Statement on Ethical Conduct in Human Research* (*2007*, *updated 2018*) (*National Health and Medical Research Council Act 1992*). Venous blood was collected from healthy adult volunteers with their informed consent, and umbilical cord blood with informed consent from mothers undergoing elective caesarean section.

All mouse cell experimental procedures, including the collection and use of murine blood and spleen, were approved by the WCHN Animal Ethics Committee and conducted in accordance to the *Australian code for the care and use of animals for scientific purposes*. Blood and spleens were collected as scavenger tissue.

### Preparation of human PBMC

PBMC were prepared by layering the samples on Ficoll® Paque Plus (GE Healthcare, Uppsala, Sweden) according to the manufacturer’s protocol. The interphase layer containing PBMC was harvested and cells were washed in RPMI-1640 medium supplemented with 2 mmol/L L-glutamine, 100 U/ml penicillin, 100 μg/ml streptomycin and 10% FCS.

### Preparation of mouse blood and splenic mononuclear cells

Scavenged spleens of adult Swiss mice were collected during training sessions from the Adelaide University Medical School animal house. The mice were given injectable anaesthesia and blood was collected by cardiac puncture. The mice were killed by cervical dislocation. Spleens were removed by dissecting the mice aseptically and placed in RPMI-1640 containing 10% FCS, penicillin (100 U/ml), streptomycin (100 µg/ml) and L-glutamine (2 mmol/l). The spleens were mashed in between the slides to prepare spleen cells. The blood or spleen cells were layered on Ficoll® Paque Plus according to the manufacturer’s protocol and after three washes, cells were used for Western blot or flow cytometry assays. Cell viability was determined by the trypan blue-exclusion method.

### Collection of cord blood samples

Cord blood samples (*n* = 9) were collected following elective caesarean sections with no complications at birth. These samples were analysed for the PKC isozymes levels within 2 hours of collection. Additionally, cord blood mononuclear cells (CBMC) were isolated by centrifugation over Ficoll® Paque Plus according to the manufacturer’s protocol and cryopreserved for later functional analysis.

### Cryopreservation of cells

Freshly isolated MC from human adult or cord blood, mouse blood or spleen were cryopreserved in freezing media containing 90% heat-inactivated FCS and 10% DMSO. Cells were incubated in a ‘Mr. Frosty’ Freezing Container (Thermo-Fisher Scientific, Scoresby Vic, Australia) overnight in a −80 °C freezer, then transferred into liquid nitrogen storage^10^.

### Western blot

Western blots were conducted essentially as described previously^9,23^. Cell lysates were prepared from either human PBMC or mouse splenic cells in a buffer containing 20 mmol/L HEPES, pH 7.4, 0.5% Nonidet P-40 (v/v), 100 mmol/L NaCl, 1 mmol/L EDTA, 2 mmol/L Na_3_VO_4_, 2 mmol/L dithiothreitol, 1 mmol/L phenylmethylsulfonyl fluoride, and 10 μg/ml of each protease inhibitor (benzamidine, leupeptin, pepstatin A, purchased from Sigma-Aldrich, St. Louis, Missouri, USA), and aprotinin (Calbiochem, Merck, Darmstadt, Germany). Lysate proteins were quantitated by using the Qubit® Protein Assay Kit (Thermo Fisher Scientific, Waltham, MA, US), prior to the addition of Laemmli buffer. The samples were boiled at 100 °C in the presence of Laemmli buffer for 5 min before loading 25 μg of sample per well in 10% SDS Stain-Free™ FastCast™ Acrylamide gels (Bio-Rad Laboratories, Hercules, California, USA) for electrophoresis using the BIO-RAD Mini-PROTEAN Tetra Cell system (Bio-Rad Laboratories) at ~175 V for 1 h. The proteins were electrophoretically transferred to nitrocellulose membrane by using a Trans-Blot® Turbo™ transfer system (Bio-Rad Laboratories). After blocking, the membranes were incubated with the appropriate mouse or rabbit monoclonal anti-PKC isozyme antibodies, individually, followed by washing and incubation with HRP-conjugated secondary rabbit anti-mouse Ig (Dako, Glostrup, Denmark), or HRP-conjugated goat anti-rabbit Ig secondary antibodies (Dako, Glostrup, Denmark) as appropriate. Immunoreactive material was detected using the Western Lightning™ Chemiluminescence Reagent Plus (Perkin Elmer, Waltham, MA) according to the manufacturer’s instructions. The protein bands on the membranes were visualized by a ChemiDoc XRS+ Imaging System and quantitated using Image Lab™ Software, Version 3.0 (Bio-Rad Laboratories). Some blots were stripped using ReBlot Plus Mild Antibody Stripping Solution (Merck-Millipore) and re-probed with mouse monoclonal GAPDH antibody (clone 71.1, Sigma-Aldrich, used at 1/15000) to confirm equal loading and protein integrity.

### Conjugation of fluorophores to the anti-PKCη, μ and βI antibodies

Conjugation of fluorophoes to anti-PKCη, anti-PKCβI and anti-PKCμ monoclonal antibodies was performed by using an Alexa Flour 488 (AF488) or AF647 labelling kit (Thermo Fisher Kit, Waltham, MA, US), and unreacted dye was removed according to the manufacturer’s instructions. Prior to conjugation, glycerol and sodium azide were removed using an Amicon Ultra-0.5 Centrifugal Filter Unit (Cat# UFC503024, Sigma Aldrich). Briefly, between 30–96 μg of an antibody was reconstituted to 0.5 ml with PBS and placed in a filter unit. Following centrifugation (14000 g × 5 min), the concentration of each antibody was adjusted to 1 mg/ml with PBS as per kit manufacturer’s instructions. The yield of labelled antibodies was around 83% of the starting material. For storage, BSA with 40% glycerol (v/v) and 0.02% sodium azide (w/v) was added such that the total protein concentration was 1 mg/ml. The antibodies were aliquoted and stored at −20 °C.

### Flow cytometric detection of PKC isozymes in whole blood

Each PKC isozyme was assessed in whole blood from healthy donors or umbilical cords, using cell surface and intracellular flow cytometry staining methods. All staining steps were performed at room temperature with incubations in the dark where possible. To 50 µl of blood per tube, varying combinations of fluorochrome-conjugated monoclonal antibodies to CD3, CD4, CD8, CD19, CD16, and CD56, were added and incubated for 15 min. This was followed by erythrocyte lysis with the addition of 2 ml of BD FACS™ Lysing Solution for a further 10 min incubation, then a centrifugation to pellet leukocytes (500 g/3 min), with the supernatant discarded. The cells were washed with 2 ml of PBS with 1% FCS, and then fixed in 250 µl of BD Cytofix/Cytoperm™ Fixation and Permeabilization Solution for 10 min. After another centrifugation and supernatant removal, the cells were permeabilised with 2 ml NET-Gel^24^ for 10 min. After supernatant removal, the fixed/permeabilised cells were Fc blocked with 1 µg of mouse/rabbit IgG for 10 min, followed by the addition fluorochrome-conjugated monoclonal antibodies to PKC isozymes or corresponding isotype control, in a final staining volume of approximately 50 µl, and incubated for 30 min. The stained cells were then washed twice with PBS + 1% FCS, and then acquired within 1 h on a BD FACSCanto, with at least 10,000 leukocyte events recorded.

PKC isozymes were assessed using FlowJo v10.1 (Ashland, Oregon, USA) on cell populations with the following gating strategy: doublets excluded using a forward scatter-height (FSC-H) versus -area (FSC-A) plot, then FSC-A versus side scatter-area (SSC-A) to ascertain lymphocyte, monocyte, and neutrophil populations. Specific lymphocyte subsets were then gated by their positivity to CD markers, or where only CD3 and CD8 were utilized in cell surface staining, CD3^+^/CD8^−^ populations were considered as CD4 helper T-cells. PKC isozymes were gated positive based on fluorescence minus one and isotype controls. The data are presented as median fluorescent intensities (MFI). Signal-to-noise was calculated as ratio of positive (PKC isozyme)/negative (isotype control) MFI, as previously described^25^.

### Maturation of cord blood T cells in culture

CBTC maturation was conducted as previously described^10^. CBMC were isolated from blood by the same process as described for PBMC isolation from adult blood and cryopreserved until assayed. Cells were thawed in a water bath and washed once with warm complete media. Viability was assessed by the trypan blue exclusion assay which revealed viability of 87–93%. CBMC cells at 1 × 10^6^/ml in complete media were seeded in a 12 well plate (total volume 2 ml). For each time point, separate wells were used. The cells were rested in a CO_2_ incubator at 37 °C for 2 h before adding phytohemagglutinin (PHA) at a final concentration of 2 μg/ml. IL-2 was added to the cells at a final concentration of 10 ng/ml on day three and media was replaced with fresh media. PKC isozymes were measured at day-0 in fresh samples and day-1 and day-7 in cultured cells.

### Statistical analyses

All data were analysed for normal distribution using the D’Agostino-Pearson and Shapiro-Wilk normality test. Multiple comparisons were performed by one-way ANOVA, followed by the Tukey multiple comparisons test. All statistical analyses were performed using GraphPad Prism version 7.02 (GraphPad Software, La Jolla, California, USA). A *P* value < 0.05 was considered statistically significant for all analyses.

## Supplementary information

Supplementary information

## Data Availability

The datasets generated during and analysed during the current study are available from the corresponding author on reasonable request.
