# Supplementary material for: Validation of monoclonal anti-PKC isozyme antibodies for flow cytometry analyses in human T cell subsets and expression in cord blood T cells
Source: Sci Rep. 2019 Jun 25;9:9263. doi: 10.1038/s41598-019-45507-2 (PMC6592917; doi:10.1038/s41598-019-45507-2)
Supplement: Supplementary file 1 — Supplementary information [file 41598_2019_45507_MOESM1_ESM.docx]

**Scientific Reports**

***Supplementary Information***

**Validation of monoclonal anti-PKC isozyme antibodies for flow cytometry analyses in human T cell subsets and expression in cord blood T cells.**

**Khalida Perveen^1,2^, Alex Quach^1,2^, Andrew McPhee^3^, Susan Prescott^4^, Simon C Barry^2^, Charles S Hii^1,2^ & Antonio Ferrante^1,2^**

^1^Department of Immunology, SA Pathology at Women’s and Children’s Hospital, North Adelaide, South Australia, Australia.

^2^The Robinson Research Institute and School of Medicine, University of Adelaide, South Australia, Australia.

^3^Department of Neonatal Medicine, Women’s and Children’s Hospital, North Adelaide, South Australia, Australia,

^4^Department of Paediatrics, University of Western Australia, Western Australia, Australia.


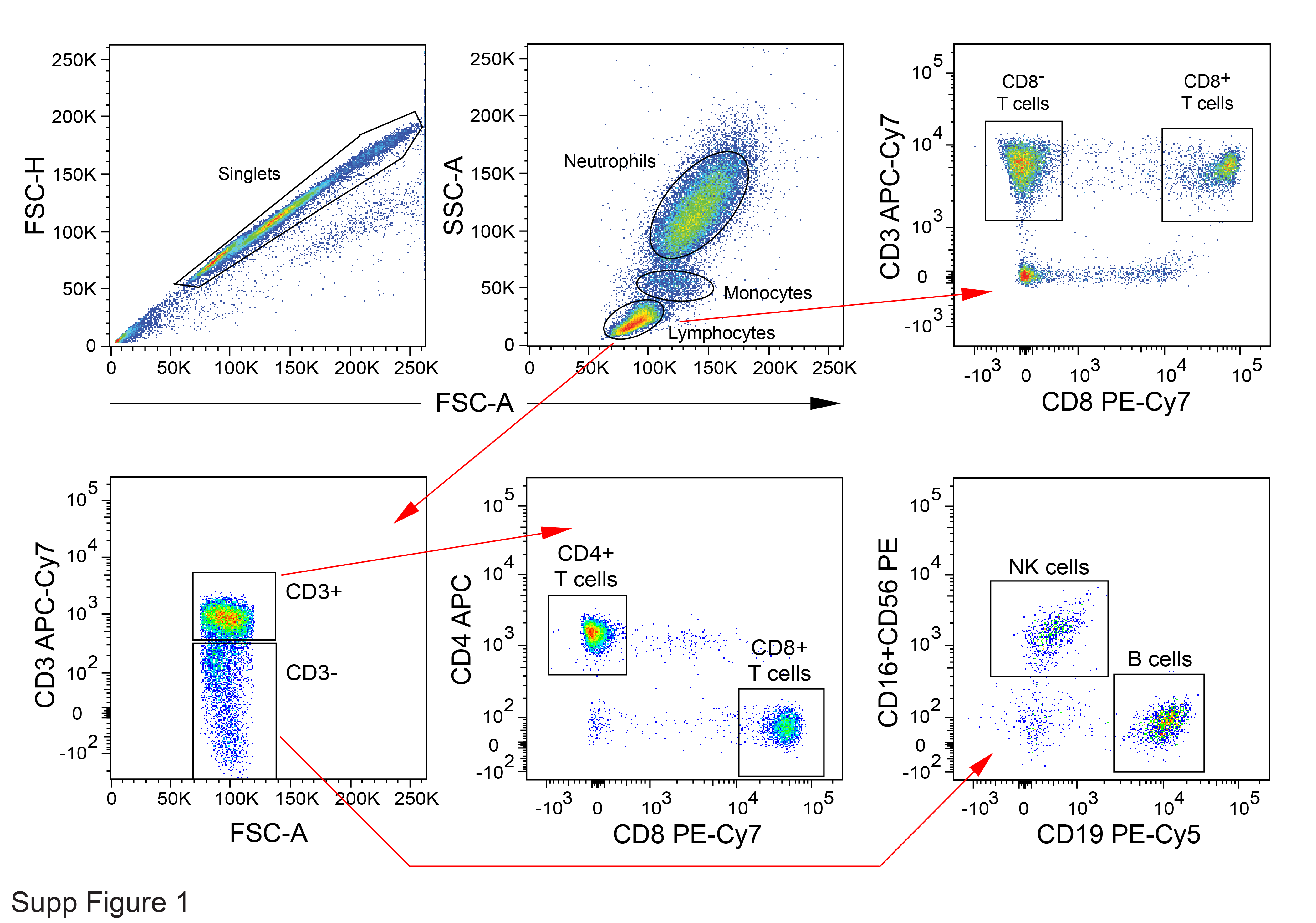


**Supplementary Figure S1.** Gating strategies for leukocyte subsets in whole blood assays for PKC detection. Following surface and intracellular staining, which included red cell lysis, leukocyte fixation and permeabilisation, cells were acquired on the flow cytometer. Singlets were gated by FSC-A/FSC-H, and SSC was used to distinguish lymphocytes, monocytes and neutrophils. The lymphocytes were further gated in either of two ways, depending on the PKC antibody panel (shown in Table S1 and S2): (1) CD4+ T cells elucidated by CD3+/CD8- gating; or (2) using CD3+ to directly detect CD4+ and CD8+ T cells, and CD3- to detect CD16+CD56+ NK cells and CD19+ B cells.


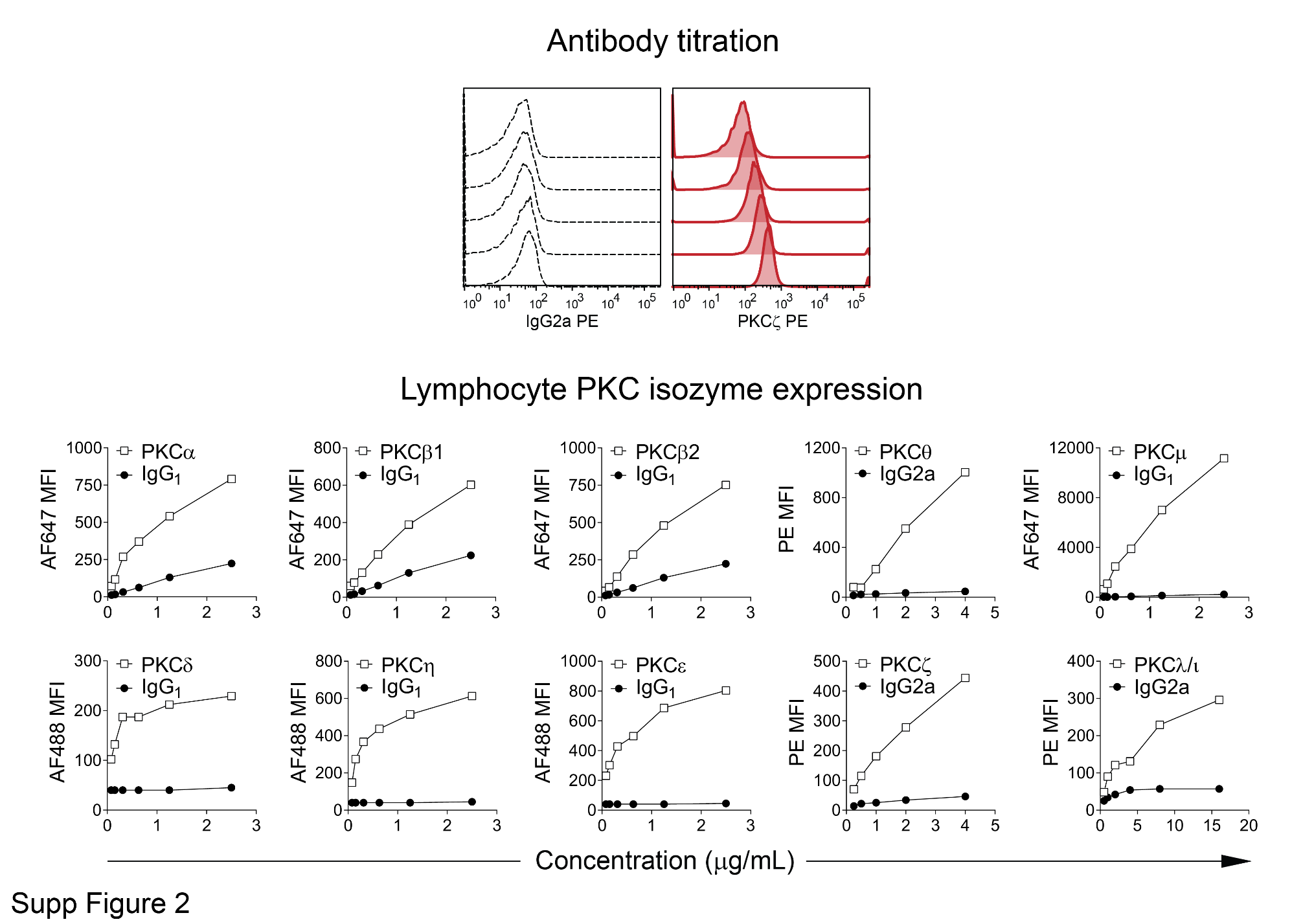


**Supplementary Figure S2.** Titration of anti-PKC isozyme antibodies and isotype control in lymphocytes. The lymphocyte fraction of whole blood was gated and assessed for staining by the anti-PKCα, β1, β, θ, δ, ε, ζ, λ/ι μ and η antibodies. The final concentration of each antibody with the respective isotype control is indicated in the graphs.


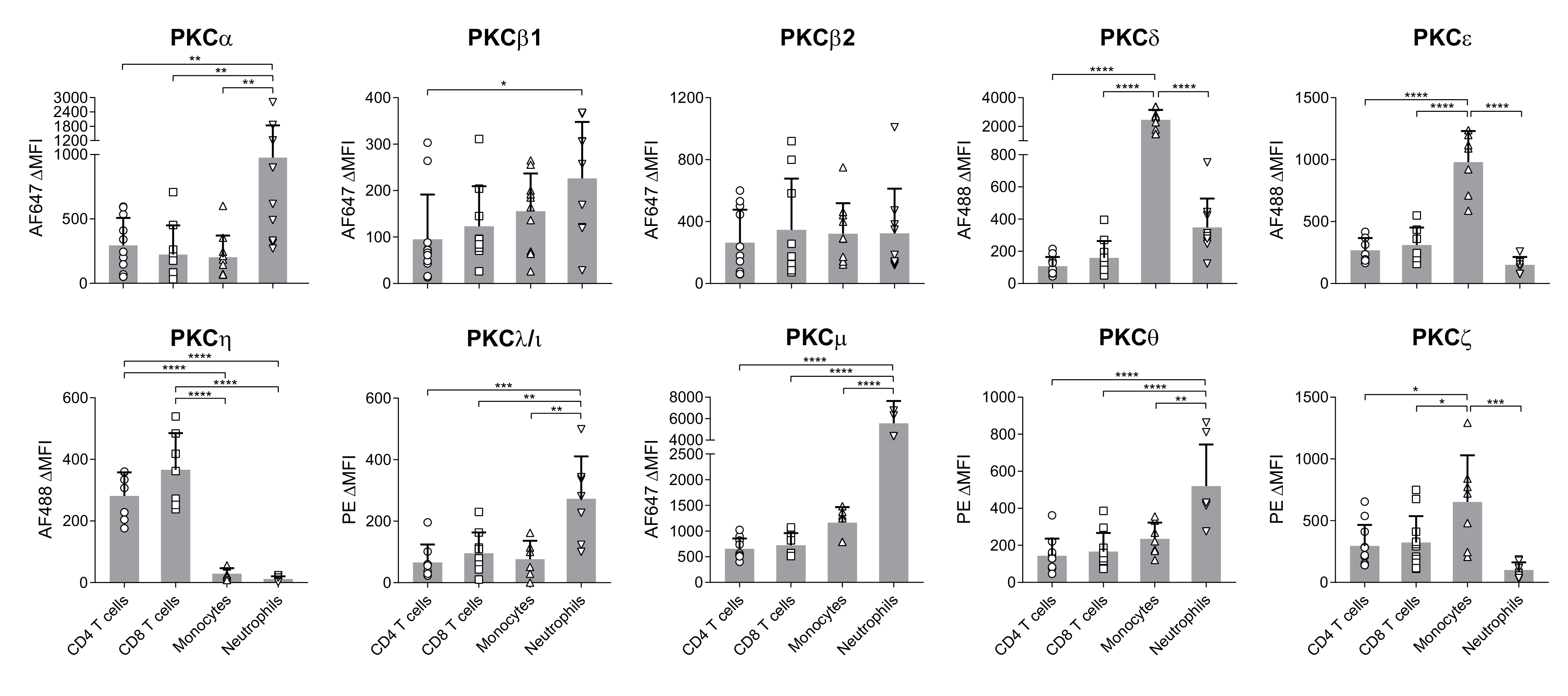


**Supplementary Figure S3.** Comparison of PKC isozymes in different cell populations of human adult whole blood by flow cytometry. Intracellular staining method was performed in fresh whole blood as per method section. PKC isozyme staining was subtracted from their corresponding isotype control staining (ΔMFI), as assessed on FSC/SSC gated lymphocytes (CD4+ or CD8+), monocytes and neutrophils. Neutrophils, *n* = 7 to 10; monocytes, *n* = 7 to 11; and CD4+ and CD8+ T cells, *n* = 11.

**
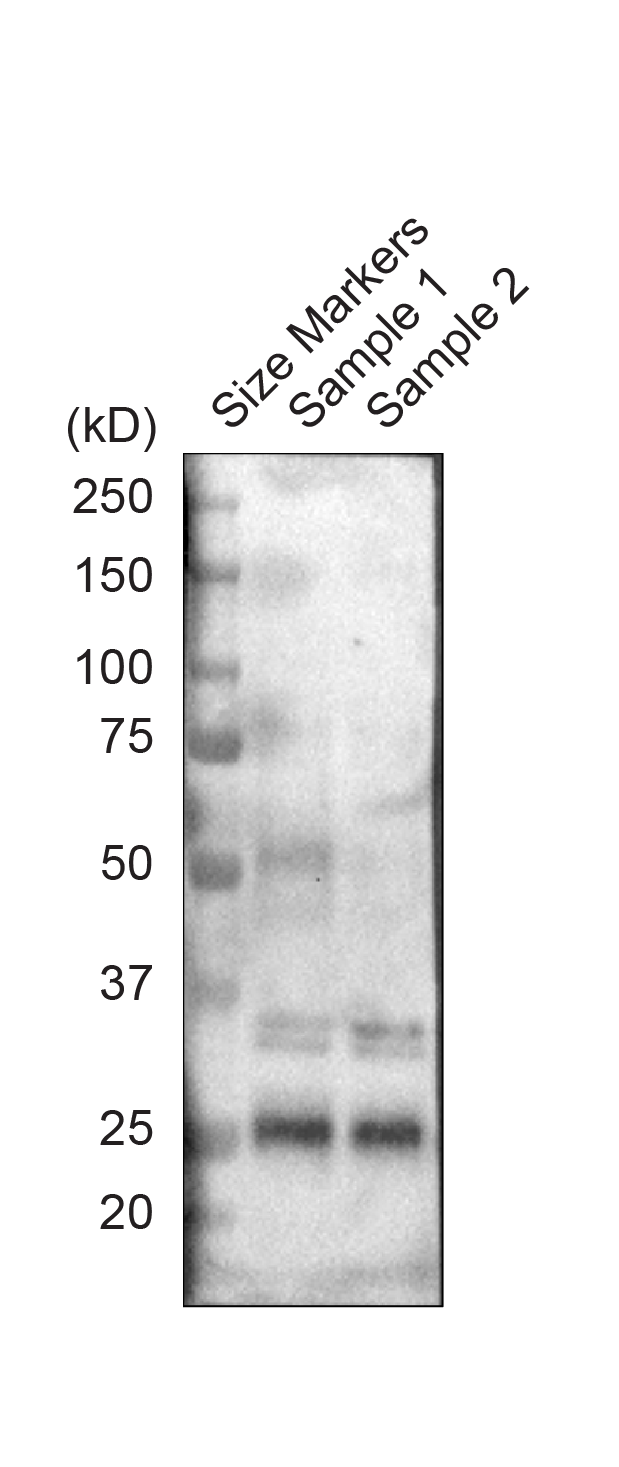
**

**Supplementary Figure S4.** Non-specific secondary antibody staining in the Western blot of murine lymphocyte lysates. Horse-radish peroxidase-conjugated rabbit anti-mouse was applied to the blot in the absence of primary antibody staining. The blot is presented intact without splicing, with lanes in their entirety.

**Supplementary Table S1. Details of antibodies used to determine expression of PKC isozymes.**

| **Name** | **Fluorochrome** | **Isotype** | **Clone** | **Company** | **Catalogue** |
| --- | --- | --- | --- | --- | --- |
| Anti-PKCα | Alexa Fluor® 647 | Ms IgG_1_, κ | H-7 | Santa Cruz Biotechnology | sc-8393 |
| Anti-PKCβII | Alexa Fluor® 647 | Ms IgG_1_, κ | F-7 | Santa Cruz Biotechnology | sc-13149 |
| Anti-PKCζ | PE | Ms IgG_2a_, κ | H-1 | Santa Cruz Biotechnology | sc-17781 |
| Anti-PKCθ | PE | Ms IgG_2a_, κ | E-7 | Santa Cruz Biotechnology | sc-1680 |
| IgG1  (isotype control) | Alexa Fluor® 647 | Ms IgG_1_, κ | MOPC-31C | BD Pharmingen™ | 566011 |
| Anti-PKCβ1* | - | Rb IgG | EPR18512 | Abcam | ab195039 |
| Anti-PKCβ1# | Alexa Fluor® 488 | Rb IgG | EPR18512 | Abcam | ab223452 |
| Anti-PKCη** | - | Rb IgG | EPR18513 | Abcam | ab179524 |
| Anti-PKCδ | Alexa Fluor® 488 | Rb IgG | EPR17075 | Abcam | ab206282 |
| Anti-PKCε | Alexa Fluor® 488 | Rb IgG | EPR1482(2) | Abcam | ab217980 |
| Anti-PKCμ* | - | Rb IgG | EP1493Y | Abcam | ab51246 |
| Anti-PKCλ/ι | PE | Ms IgG_2a_, κ | H-12 | Santa Cruz Biotechnology | sc-17837 |
| Anti-CD3 | PE-Cy™5 | Ms IgG_2a_, κ | HIT3a | BD Pharmingen™ | 555341 |
| Anti-CD3 | APC-H7 | Ms IgG_1_, κ | SK7 | BD Pharmingen™ | 560176 |
| Anti-CD8 | PE-Cy™7 | Ms IgG_1_, κ | RPA-T8 | BD Pharmingen™ | 557746 |
| Rabbit (DA1E) mAb IgG XP® Isotype Control | Alexa Fluor® 488 | Rb IgG | - | Cell Signalling Technology | 2975 |
| Rabbit (DA1E) mAb IgG XP® Isotype Control | Alexa Fluor® 647 | Rb IgG | - | Cell Signalling Technology | 2985 |
| Mouse IgG (isotype control) | PE | Ms IgG_2a_, κ | X39 | BD | 340459 |
| Mouse IgG - Isotype Control | - | Ms polyclonal IgG | - | Abcam | ab37355 |
| Rabbit IgG Isotype Control | - | Rb IgG | - | Invitrogen | 02-6102 |

*Labelled with Alexa Fluor® 647 Protein Labelling Kit (Invitrogen, Catalogue # A20173).

**Labelled with Alexa Fluor® 488 Microscale Protein Labelling Kit (Invitrogen, Catalogue # A30006).

#Used in PKC levels in cord blood and in kinetic experiments only.

**Supplementary Table S2. Staining panel for the determination of PKC isozymes in peripheral blood subsets.**

| **Target** | **Fluorochrome** | **Clone** |
| --- | --- | --- |
| Anti-PKCα | AF647 | H-7 |
| Anti-PKCβII | AF647 | F-7 |
| Anti-PKCβ1 | AF647 | EPR18512 |
| Anti-PKCζ | PE | H-1 |
| Anti-PKCθ | PE | E-7 |
| Anti-PKCλ/ι | PE | H-12 |
| Anti-PKCη | AF488 | EPR18513 |
| Anti-PKCδ | AF488 | EPR17075 |
| Anti-PKCε | AF488 | EPR1482(2) |
| Anti-PKCμ | AF647 | EP1493Y |
| Mouse IgG1k Isotype control | AF647 | - |
| Rabbit mAb IgG Isotype Control | AF488 | - |
| Rabbit mAb IgG Isotype Control | AF647 | - |
| Mouse mAb IgG2ak | PE | - |
| Anti-CD3 | APC-H7 | SK7 |
| Anti-CD8 | PE-CY7 | RPA-T8 |

**Supplementary Table S3. Staining panel for the determination of PKCη in lymphocyte subsets.**

| **Target** | **Fluorochrome** | **Clone** |
| --- | --- | --- |
| Anti-PKCη | AF488 | EPR18513 |
| Anti-PKCε | AF488 | EPR1482(2) |
| Rabbit mAb IgG Isotype Control | AF488 | - |
| Anti-CD3 | APC-_CY_7 | SK7 |
| Anti-CD8 | PE-_CY_7 | RPA-T8 |
| Anti-CD4 | APC | RPA-T4 |
| Anti-CD19 | PE-_CY_5 | HIB19 |
| Anti-CD16 | PE | 3G8 |
| Anti-CD56 | PE | N901(HLDA6) |
